# RGB-D salient object detection via convolutional capsule network based on feature extraction and integration

**DOI:** 10.1038/s41598-023-44698-z

**Published:** 2023-10-17

**Authors:** Kun Xu, Jichang Guo

**Affiliations:** 1 School of Electrical and Information Engineering, Tianjin University, Tianjin, 300000 People’s Republic of China; 2 Key Laboratory of Computing Power Network and Information Security, Ministry of Education, Shandong Computer Science Center (National Supercomputer Center in Jinan), Qilu University of Technology (Shandong Academy of Sciences), Jinan, 250014 People’s Republic of China; 3 Shandong Provincial Key Laboratory of Computer Networks, Shandong Fundamental Research Center for Computer Science, Jinan, China

**Keywords:** Electrical and electronic engineering, Computer science

## Abstract

Fully convolutional neural network has shown advantages in the salient object detection by using the RGB or RGB-D images. However, there is an object-part dilemma since most fully convolutional neural network inevitably leads to an incomplete segmentation of the salient object. Although the capsule network is capable of recognizing a complete object, it is highly computational demand and time consuming. In this paper, we propose a novel convolutional capsule network based on feature extraction and integration for dealing with the object-part relationship, with less computation demand. First and foremost, RGB features are extracted and integrated by using the VGG backbone and feature extraction module. Then, these features, integrating with depth images by using feature depth module, are upsampled progressively to produce a feature map. In the next step, the feature map is fed into the feature-integrated convolutional capsule network to explore the object-part relationship. The proposed capsule network extracts object-part information by using convolutional capsules with locally-connected routing and predicts the final salient map based on the deconvolutional capsules. Experimental results on four RGB-D benchmark datasets show that our proposed method outperforms 23 state-of-the-art algorithms.

## Introduction

With the popularity of Microsoft Kinect, Intel RealSense and some modern smartphones (e.g. iPhone X, and Samsung Galaxy S20), depth images can be obtained easily and conveniently. As a result, Salient Object Detection (SOD) by using RGB images and depth images (RGB-D images) has become a hot research topic. Benefiting from its stable geometry and additional contrast cues, depth images can provide important complementary information for SOD. Especially, the emergence of Fully Convolutional Neural Networks (FCNs) makes it possible to capture multi-level and multi-scale features, thereby boosting the performance of RGB-D SOD^1–9^.

**Figure 1:**
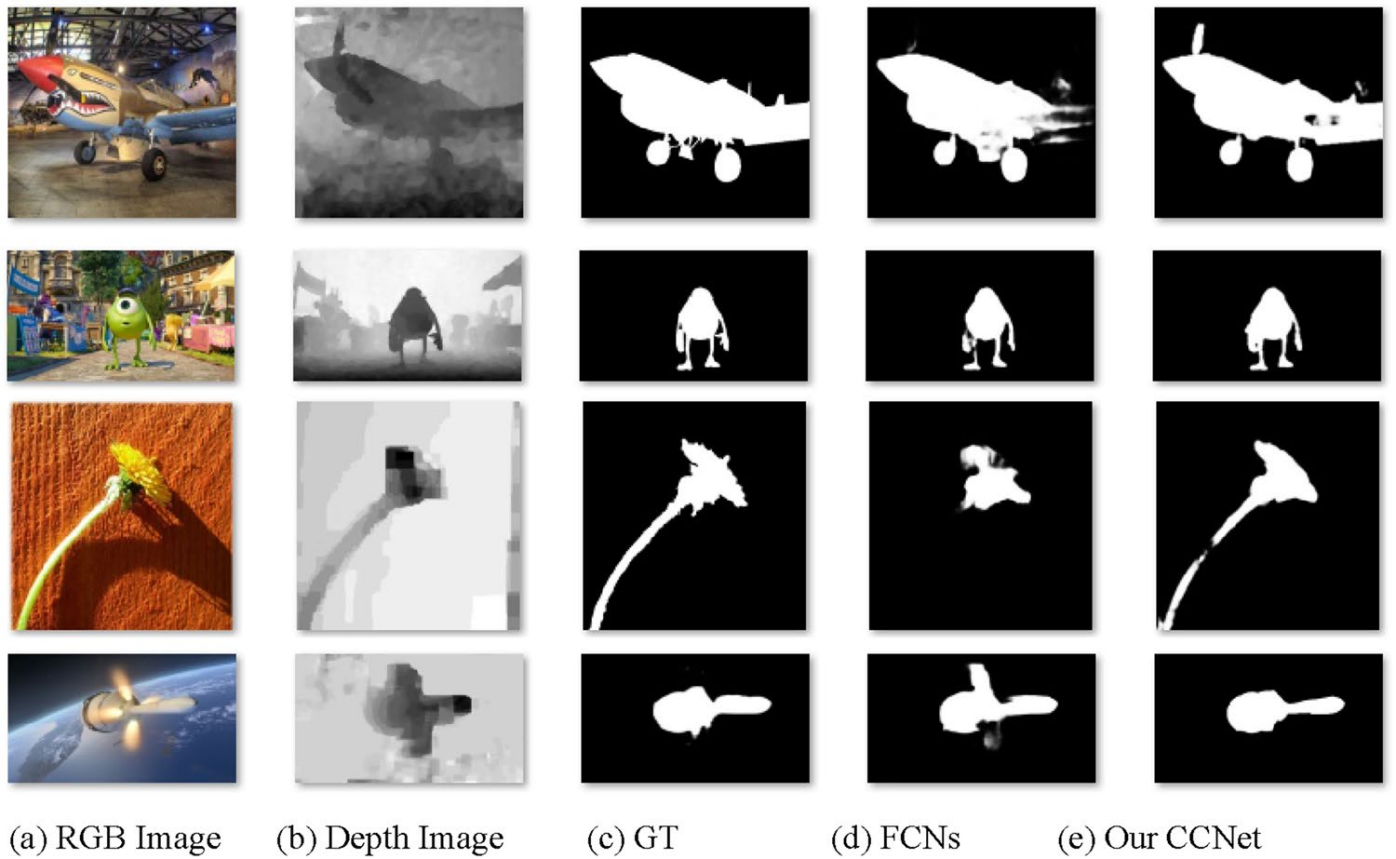
The examples of object-part relationship for SOD. For the first row, the tail wing of an aircraft is not recognized as the salient object by FCNs while our proposed method predicts the aircraft as a whole. In the second row, there is an incoherence in the right arm of the cartoon figure predicted by the FCNs while the proposed method regards the right arm and the cartoon figure as an integral whole. In third line, the salient object predicted by FCNs misses the stem of the plants while the flower and the stem are predicted by our CCNet. For the last row, the flame of the satellite is misidentified as the salient object by the FCNs. However our proposed method suppresses the interference by identifying the satellite as a complete object. *Note*: Reproduced with permission of references 25, Copyright of ©2017 IEEE, references 26, Copyright of ©2016 IEEE, references 27, Copyright of ©2018 IEEE, references 28, Copyright of ©2015 IEEE.

Most FCNs predict the salient object by assembling multi-level features. However, there is an object-part dilemma under the mechanism of FCNs, which is demonstrated in Fig. 1 with four representative examples. As shown in Fig. 1d, some parts of the predicted salient object from FCNs are immersed in the background or disturbed by the background, they may be easily mislabeled as non-salient regions. It results in incomplete segmentation. In other words, the relationships between an object and its parts are not taken into consideration by existing FCNs. Ideally, a salient object is a complete entity, which is composed of several associated parts. If a large proportion of the object were predicted as the salient region, the complete object would be determined as a salient object. As shown in Fig. 1e, the salient objects are segmented as a whole with a high probability when the object-part relationship is taken into consideration by the Capsule Network (CapsNet).

Recently, the CapsNet^10–12^ has been proposed to preserve vector quantity, rather than scalar quantity, by replacing max-pooling operation with convolutional strides and dynamic routing. The vector quantity is capable of preserving object-part relationships, which is the basic element of a capsule. A capsule encapsulates a group of neurons whose outputs are vector quantity, representing different properties of an entity, including position, size, sharp and orientation, which preserve enough information to explore the object-part relationship. Furthermore, associated parts of an object are represented by child capsules. Then, children capsules are clustered by the dynamic routing algorithm to generate parent capsules. Unfortunately, despite the great performance, CapsNet is known for its high computational demand, both in terms of memory and run-time, even for a very simple image classification task. Especially, child capsules store all intermediate representations and parent capsules are clustered by the dynamic routing algorithms, which determines coefficients between every child capsule and every parent capsule. A large amount of GPU memory required, when the dynamic routing algorithms occurs. Therefore, it is impractical for CapsNet to deal with SOD.

Inspired by these observations, we introduce object-part relationship for RGB-D SOD in this paper, which provides a solution to incomplete salient object segmentation. A Convolutional Capsule Network based on Feature Extraction and Integration (CCNet) for RGB-D SOD is proposed to explore the object-part relationship with low computation demand. Our system consists of two key parts. One is proposed to extract and integrate features based on VGG, Global Context Module(GCM)^13^, attention mechenism^14, 15^ and FDM(Feature Depth Module). The other one is the Feature-integrated Convolutional Capsule Network (FiCaps), whose structure is composed of the convolutional part and deconvolutional part, similar to SegCaps^16^. Specifically, in our proposed FiCaps, child capsules are only routed to parent capsules within a defined local kernel. Besides, the transformation matrices are shared for each member of the grid within a capsule.

Our contributions are summarized as follows:

1. We introduce the object-part relationship into RGB-D SOD by using the CCNet. To the best of our knowledge, this is an earlier attempt to apply CapsNet to explore object-part relationships for RGB-D SOD.
2. A novel FiCaps is proposed to integrate external multi-level features with internal capsules. As demonstrated in Fig. 1e, our proposed method can recognize and segment the salient object as a whole with a high probability, compared with the methods based on FCNs.
3. We compare our approach with 23 state-of-the-art RGB-D SOD. The experimental results demonstrate that our CCNet outperform other state-of-the-art algorithms.

## Related works

The utilization of RGB-D images for SOD based on FCNs has been extensively explored for years. Based on the goal of this paper, we review the RGB-D SOD methods as well as the CapsNet and illustrate the differences between our proposed methods and related works.

### RGB-D salient object detection

The pioneering work was produced by Niu et al.^17^ based on traditional methods. After that, various hand-crafted features originally applied for RGB SOD were extended to RGB-D SOD, such as^18, 19^. In this paper, we pay much attention to the RGB-D SOD based on deep learning algorithms. For example, Xu et al.^20^ propose a lightweight SOD for real-time localization which is composed of lightweight feature extraction network based on multi-scale attention, the jump connections and a residual refinement module. Chen and Fu^20^ propose an alternate refinement strategy and combine a guided residual block to predict refined features and salient maps, simultaneously. Lei^22^ proposes SU2GE-Net. Firstly, the CNN-based backbone is replaced by the transformer-based Swin-TransformerV2. Besides, an edge-based loss and training-only augmentation loss are introduced to enhance spatial stability. Zhao et al.^23^ build a real single-stream network by combining RGB-D images at the starting point, taking advantage of the potential contrast information provided by depth images. Compared with the above algorithms, there are similarities and differences. For the similarities, the design idea between our proposed algorithms and SU2GE-Net in the related work is the same. Both of them try to replace the CNN-based backbone with a novel backbone, such as Swin-TransformerV2 or CapsNet. Furthermore, the structure of salient object detectors is the same as well, including the encoders and decoders. For the differences, the CCNet predicts the salient object mainly based on CapsNet, whose basic elements are vector quantity. However, other salient detectors are scalar quantity.

### Capsule network

Recently, a novel deep learning network, named CapsNet, was developed by Hinton et al. ^10^. A capsule is essentially a group of neurons, which represent a specific type of the entity, such as position, size, orientation, deformation, texture and etc. The CapsNet is totally different with the FCNs in two aspects. On the one hand, neurons of FCNs are scalars while that of CapsNet are vectors. On the other hand, the FCNs extract and integrate multi-level features by encoder and decoder while the CapsNet matches associated active child capsules into parent capsules by dynamic routing algorithm. Then, Sabour et al.^11^ proposes the vector CapsNet. An iterative dynamic routing algorithm was proposed to assign child capsules to corresponding parent capsules via transformation weights. The spatial relationship between a part and a object is encoded and learned by the dynamic routing algorithm and transformation weights. One year later, Hinton et al.^12^ consolidated the vector CapsNet by proposing a matrix CapsNet, whose capsule is composed of a pose matrix and an activation probability. The coefficients between the child capsule and the parent capsule are calculated by the iterative Expectation–Maximization (EM) algorithm, by finding the tightest clusters of capsules. Compared with the vector CapsNet, the transformation matrix of the matrix CapsNet has much less parameters. Furthermore, the matrix CapsNet use the iterative EM to measure the similarities between capsules, while the vector CapsNet uses the cosine similarity. In the view of its advances, some attempts have been made to apply CapsNet for several computer vision tasks, including the object segmentation and SOD. To reduce the high computational demand, LaLonde and Bagci design the SegCap^16^ based on the vector Capsules to solve the object segmentation. It extends the idea of convolutional capsules with the locally-connected routing and the concept of deconvolutional capsules. Liu and his colleague^24^ propose the Two-Stream Part-Object Relational Network (TSPORTNet) to implement the matrix CapsNet for SOD, whose activation map is the final salient map. Both methods try to reduce the computation demand, which make them possible to be used in large-scale image tasks. In this paper, the structure of our proposed method is the similar to that of SegCaps. Different from the SegCaps, our proposed method in the encoder excavate capsules and concatenates them with corresponding multi-level features. However, the encoder and decoder of the SegCaps are enclosed environment. Furthermore, the TSPORTNet prefers to explore the object-part relationship based on the matrix Capsules and use the activation map as the salient map. The predicted salient map is coarse and needs to be refined. On the contrary, our proposed FiCaps uses extracted features from FCNs as the input. Subsequently, a refined salient map is predicted directly by FiCaps, without post-processing.

## Methodology

**Figure 2:**
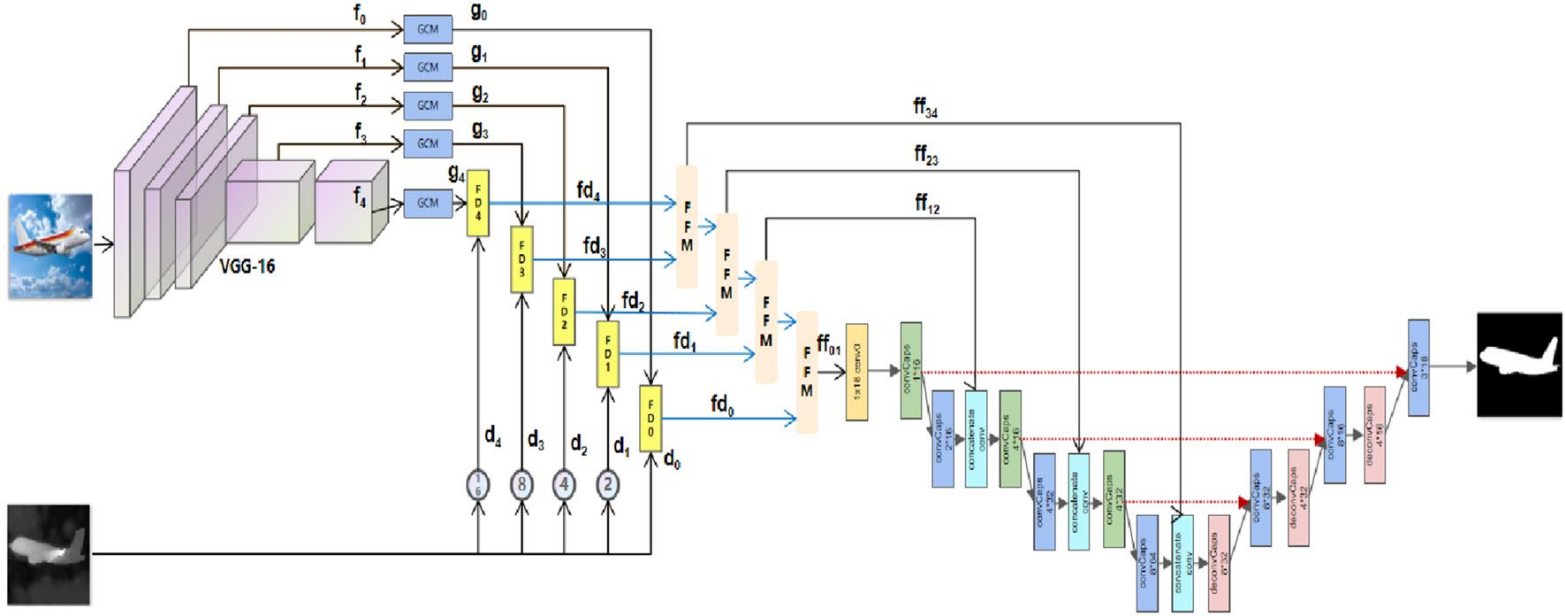
The framework of CCNet. The features are extracted by the VGG backbone, which is denoted as $\left( {f_{0} ,f_{1} ,f_{2} ,f_{3} ,f_{4} } \right)$. The features $\left( {g_{0} ,g_{1} ,g_{2} ,g_{3} ,g_{4} } \right)$ refer to the outputs of the GCM. The depth image is downsampled directly which is labeled as $\left( {d_{0} ,d_{1} ,d_{2} ,d_{3} ,d_{4} } \right)$. Then, the features from GCM and depth images are integrated by FDM, whose outputs are $\left( {fd_{0} ,fd_{1} ,fd_{2} ,fd_{3} ,fd_{4} } \right)$. In the next step, the outputs of FDM are aggregated by Feature Fusion Module(FFM) progressively, denoted as $\left( {ff_{01} ,ff_{12} ,ff_{23} ,ff_{34} } \right)$.In FiCaps, the *conv* means the traditional convolution with 1 × 1 kernel size. The *convCaps* means the convolution capsule layer, whose stride and padding is equal to 1 or 2. The *deconvCaps* refers to the deconvolution capsule layer, implemented by transposed convolution with stride 2 and padding 2. The concatenation indicates a series of operations, including concatenation, reshape and convolution, for integrating internal capsules and external features.

This paper begins by demonstrating an overall architecture of CCNet, which is depicted in Fig. 2. It will then go on to introduce their principles and detail information of modules.

### Overall architecture

Figure 2 shows the overall architecture of CCNet. Our system begins with a VGG backbone, extracting multi-level features. Then, these features are input into GCM to further exploit. The depth image is downsampled by the max pooling to shrink it by a corresponding multiply, including 2, 4, 8, 16, respectively. In the next step, depth images are integrated with features from GCM by FDM directly, based on attention mechanism^14, 15^. After that, the outputs of FDM are integrated by FFM progressively, whose outputs are further input into FiCaps to fuse external multi-level features with capsules ulteriorly. The structure of FiCaps is similar to U-Net. In the encoder, these capsules are processed by convolutional capsule layers, which map the child capsules to the parent capsules by the dynamic routing algorithms in defined local connections. Besides, the concatenation module in the encoder is proposed to integrate external features with internal capsules. When it comes to the decoder, the capsules are processed by the deconvolutional capsule layers which are mainly composed of transposed convolution with stride 2. Finally, the output of FiCaps is the predicted salient map.

### Feature depth module

**Figure 3:**
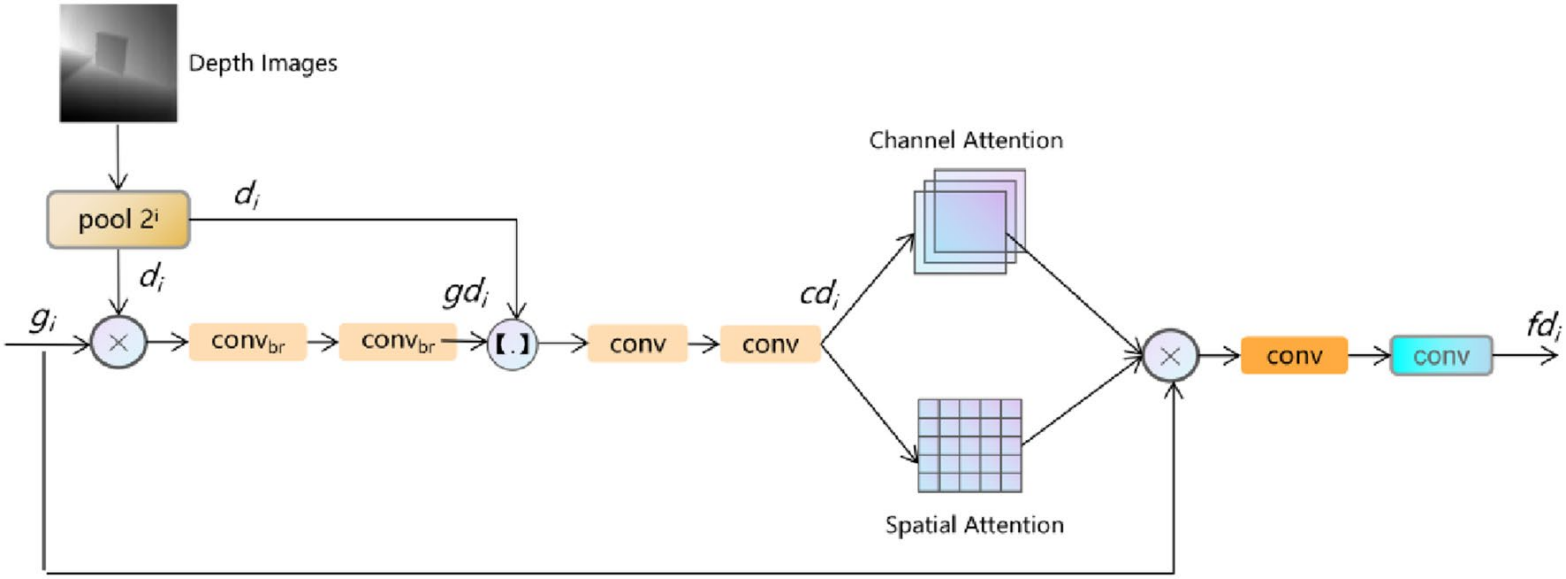
The structure of FDM. The *conv*_*br*_ refers to the 3 × 3 convolution with batch normalization and relu operation while the *conv* means the 3 × 3 convolution. The symbol *X* and *[.]* indicates the multiplication and the concatenation operation in the pixel level. The *pool* refers to the pooling operation, whose multiple is 2 to i.

The FDM is used to reweight features from GCM based on depth images. The structure of GCM is introduced in^13^ in details. In Fig. 3, we multiply features with the depth image which is downsampled to the corresponding size. Then, the product are processed by two convolutions with batch normalization and Relu operation. Consequently, these features are concatenated with downsampled depth images and are processed by two convolutions. Next, we facilitate the attention mechanism, including the channel attention and the spacial attention, to generate a reweighted map and multiply it with input features *g*_*i*_, following convolutions. The procedure is formulated as follows:

1 $$gd_{i} = conv_{br} \left(conv_{br} \left( {g_{i} *d_{i} } \right)\right)$$

2 $$cd_{i} = conv\left( {conv\left( {cat\left( {gd_{i} ,d_{i} } \right)} \right)} \right)$$

3 $$fd_{i} = conv\left( {conv_{br} \left( {g_{i} *CA\left( {cd_{i} } \right)*SA\left( {cd_{i} } \right)} \right)} \right)$$

where $g_{i}$ and $d_{i}$ refers to the *i*th feature from GCM and depth image with 2^i^ times downsampling. The $conv_{br}$ and $conv$ indicates the 3 × 3 convolution with and without the batch normalization and relu operation. The *CA* and *SA* means the channel attention and the spacial attention. The parameter *i* ranges from 1 to 4. The symbol $*$ means the multiplication operation in the pixel level.

### Feature fusion module

The FFM integrates adjacent two features from high-level to low-level, generating the feature map. As showed in Eq. (4), two input features first undergo the convolution layers, respectively. Then, the relative high-level feature is upsampled and concatenated with the low-level feature, which is further processed by two convolution layers.

4 $$ff_{i,i - 1} = conv \left(conv \left(cat \left(up \left(conv_{a} \left( {fd_{i} } \right)\right),conv_{b} \left( {fd_{i - 1} } \right)\right)\right)\right)$$

where $fd_{i}$ and $fd_{i - 1}$ refers to the relative high-level feature and low-level feature, respectively. The $conv_{a}$, $conv_{b}$ and $conv$ all indicate the convolution with batch normalization and relu operation. The $up$ indicates the 2-times upsample operation and the $cat$ means the concatenation operation. The *i* ranges from 1 to 4.

### Feature-integrated convolutional capsule network

**Figure 4:**
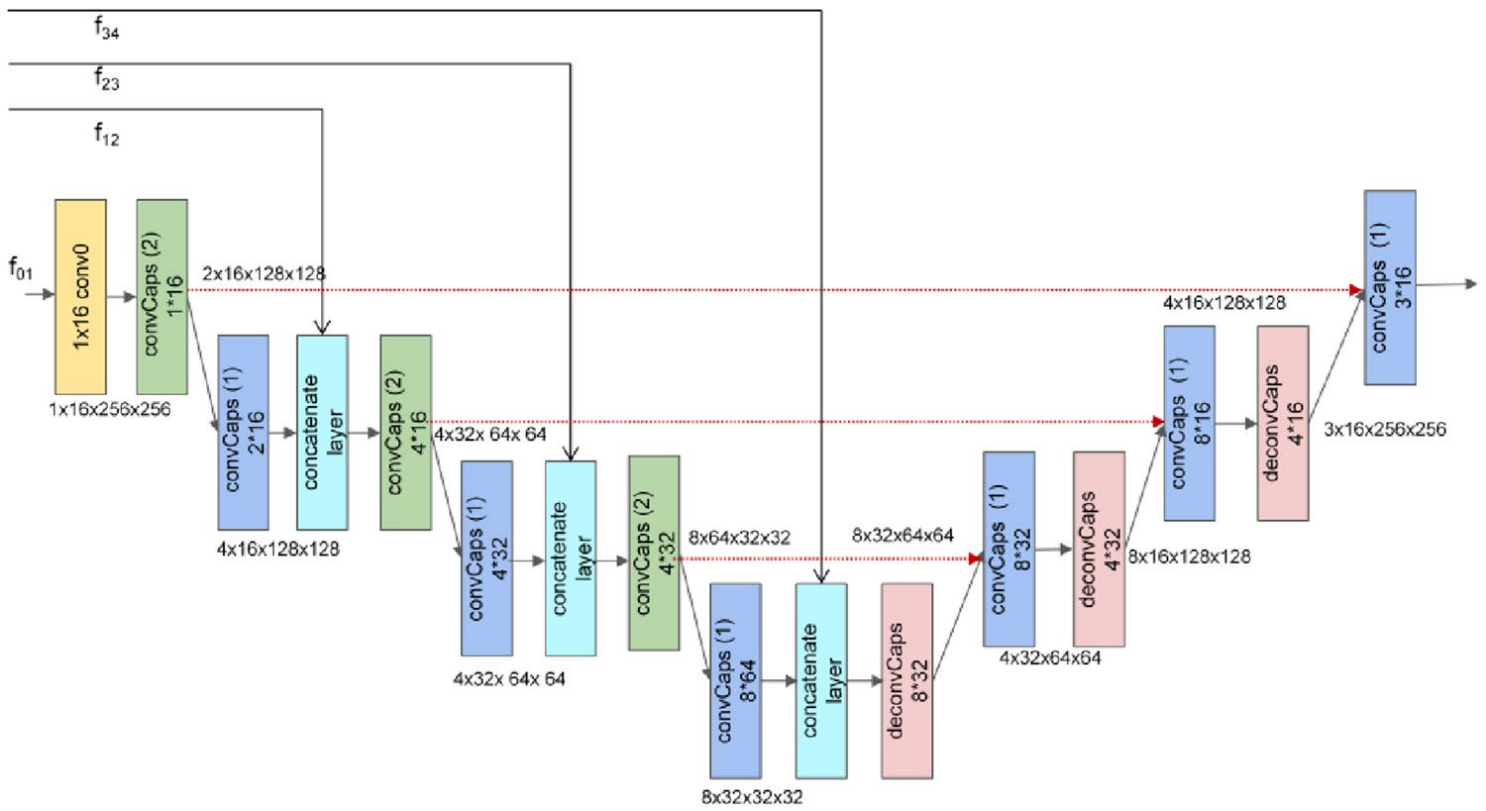
The structure of FiCaps. The *conv0* represents the traditional 1 × 1 convolution. The *convCaps(2)* indicates the convolutional capsule layer with stride 2 while the *convCaps(1)* means the convolutional capsule layer with stride 1. The *deconvCaps* represents the deconvolutional capsule layer based on the transposed convolution. The *red dash arrow* refers to the bridge connection between the capsule in the encoder and the corresponding capsule in the decoder. The *black arrow* is the data flow whose data size is described by the text near it. The *concatenate layer* means the concatenation operation for integrating the internal capsules with the external features. The *f*_*01*_, *f*_*12*_, *f*_*23*_ and *f*_*34*_ means the corresponding external features.

Figure 4 shows the details of FiCaps. We first introduce the structure of FiCaps and then elaborate the detail, including the convolutional capsule layer, the deconvolutional capsule layer and the concatenation layer. The FiCaps shares the same architecture with the U-Net. For the encoder, it contains two basic modules, the convolutional capsule layer and the concatenation layer. In the decoder, it is composed of the convolutional capsule layer and the deconvolutional capsule layer. First of all, the feature map from FFM is transformed into the capsule. Then, the capsule (1 × 16 × 256 × 256) is downsampled by a convolutional capsule layer with stride 2, which is further put into a convolutional capsule layer with stride 1, for mapping the child capsule to the parent capsule by using the dynamic routing algorithm. Subsequently, the concatenation layer first transforms the capsule (4 × 16 × 128 × 128) back to feature map (64 × 128 × 128) via reshape operation. Then, the transformed feature map is concatenated with the corresponding external features (32 × 128 × 128), which is then processed by the convolution and reshaped into the capsule (4 × 16 × 128 × 128). Such procedure is executed three times until the capsule (8 × 32 × 32 × 32) is obtained. In the decoder, the capsule is first upsampled by a deconvolution capsule layer with stride 2. Then, the upsampled capsule (8 × 32 × 64 × 64) and the corresponding capsule in the encoder are concatenated by the bridge connection to generate the capsule (8 × 32 × 64 × 64), which is then processed by the convolutional capsule layer. As well, such procedure is repeated three times to predict the final salient map.

### Convolutional and deconvolutional capsule layer

Both convolutional and deconvolutional capsule layer contain two parts. One is the transformation module of the capsule and the other one is the dynamic routing algorithm. There are seven parameters in a capsule layer, which can be described as $capsule layer\left( {in, inv, op, s, on, onv, rt} \right)$.The *in* and *inv* means the number of input capsule and the number of vector of input capsule while the *on* and *onv* means the number of output capsule and the number of vector of output capsule, respectively. There are two options of *op*, including *‘conv’* and *‘deconv’*. When the *op* is *‘conv’*, it means the convolution capsule layer. When the *op* is ‘*deconv’*, it means the deconvolutional capsule layer. The *s* refers to the number of stride in the convolution, cooperating with the *op* to accomplish different operations. If *op* is *‘conv’* and *s* is 2, it means a convolution with 2 times downsampling. Furthermore, if *op* is *‘deconv’* and *s* is 2, it means a convolution with 2 times upsampling. The *rt* means the iteration time of dynamic routing algorithm, which is set to 3 in this paper.

The convolutional capsule layer decides how to assign active child capsules to parent capsules. This is similar to the process of clustering. Each relatively parent capsule corresponds to a cluster center and each relatively child capsule corresponds to a data point, which can be solved by an EM algorithm. This mapping is measured by a transformation matrix, called voting in EM routing and defined as:

5 $$V_{ij}^{\left( l \right)} = c_{i}^{\left( l \right)} T_{ij}^{\left( l \right)}$$

where $c_{i}^{\left( l \right)}$ and $c_{i}^{{\left( {l + 1} \right)}}$ refers to the child capsule and the parent capsule. $V_{ij}^{\left( l \right)}$ means the voting result from capsule *i* at layer *l* for capsule *j* at layer *l* + *1.* $T_{ij}^{\left( l \right)}$ is a transformation matrix. Next, a Gaussian mixture model is introduced. Supposing Gaussian distribution $N\left( {x; \mu , \Sigma } \right)c_{i}^{\left( l \right)}$ has a diagonal covariance matrix *diag(*$\sigma^{2}$*)*. The posterior probability of a $V_{ij}^{\left( l \right)}$ belonging to the *j*th Gaussian is defined as:

6 $$R_{ij} = \frac{{a_{i} N\left( {V_{ij} ;\mu_{j} ,diag\left( {\sigma_{j}^{2} } \right)} \right)}}{{\mathop \sum \nolimits_{j} a_{i} N\left( {V_{ij} ;\mu_{j} ,diag\left( {\sigma_{j}^{2} } \right)} \right)}}$$

where activation *a*_*j*_ for capsule j is a mixture coefficient of Gaussian mixture model and *V*_*ij*_ is treated as a k*d’-dimensional vector. As a result, the child capsules vote for the parent capsule *j*, the contribution coefficient *r*_*ij*_ of capsule *i* when calculating cluster center (capsule) *j* should consider its activation value *a*_*i*_ as follows:

7 $$r_{ij} = \frac{{a_{i} R_{ij} }}{{\mathop \sum \nolimits_{i} a_{i} R_{ij} }}$$

Finally, the procedure of the convolutional and deconvolutional capsule layer is discussed as follows. First and foremost, we transform capsules to feature maps, by reshaping capsules *[n, in, inv, h, w]* into feature maps [n, in * inv, h, w], following the convolution layer. Then, we transform feature maps back to capsules, reshaping feature maps [n, on * onv, h, w] into capsules *[n, on, onv, h, w]*. Finally, the capsules execute the dynamic routing by using the EM algorithm with *r* times.

### Concatenation layer

The concatenation layer includes two reshape operations, a concatenation operation and a convolution. Supposing the size of capsule from convolutional capsule layer is *[b, c, v, h, w]* and the size of external feature map is *[b, n, h, w]*. The procedure of the concatenation layer can be discussed as follow. In the first stage, we transform capsules to feature maps, by reshaping capsules *[b, c, v, h, w]* into feature maps [n, c * v, h, w]. Therefore the shape of capsules and features maps is the same. Furthermore, we concatenate the transformed feature maps with the external features, following the convolutional layer. Lastly, the concatenated result transforms back to the capsules, by reshaping the feature maps [n, c * v, h, w] back into capsules *[b, c, v, h, w]*.

### Loss function

The parameters of our proposed method are supervised by the cross-entropy loss and the margin loss, which are described as:

8 $$Loss = \alpha \cdot CE + \beta \cdot ML$$

9 $$CE = - (gt \cdot log\left( {pred} \right) + \left( {1 - gt} \right) \cdot log\left( {1 - pred} \right)$$

10 $$ML = gt \cdot max\left( {0, m^{ + } - pred} \right) + 3 \cdot \left( {1 - gt} \right) \cdot max\left( {0,pred - m^{ - } } \right)$$

where *CE* and *ML* represents the cross entropy loss and margin loss, respectively. The *gt* and *pred* indicates the ground truth and the prediction of salient object. The *m*^+^ and *m*^*-*^ refers to the constant parameter in this paper, which is set to 0.9 and 0.1, respectively. The $\alpha ,\beta$ are set to 1.

## Experiment and analyze

In this section, numerous experiments are conducted to verify the effectiveness and superiority of CCNet and modules, evaluating by four evaluation metrics.

### Benchmark datasets and evaluation metrics

We evaluate the performance of our model on four public RGB-D benchmark datasets. NJU2K^25^ (1985 samples), NLPR^26^ (1000 samples), STERE^27^ (1000 samples) and SIP^28^ (929 samples). We choose the same 700 samples from NLPR and 1500 samples from NJU2K to train our algorithms. The remaining samples are used for testing.

Four widely-used metrics are used to evaluate the performance, including Mean Absolute Error (MAE), F-measure (${\text{F}}_{{{\upbeta } - {\text{max}}}}$)^29^, S-measure (${\text{S}}_{{\upalpha }}$)^30^, E-measure (${\text{E}}_{{\upxi }}$)^31^.

### Implementation details

Our proposed CCNet is implemented in Pytorch, which is trained for 300 epochs on a single NVIDIA Tesla T4 GPU. The Adam optimizer is used with default values. The initial learning rate is set as 1e−4 for Adam optimizer and the batch size is 10. The poly learning rate policy is used, where the power is set to 0.9. For the data augment, every input data batch in the training session are resized to 256 × 256 with random flipping, rotation, color enhance and random pepper. In the training session, the RGB images, depth images and GT are combined together as data batch. During the inference session, RGB-D images are put into the trained model to predict the salient map, without any other post-processing.

### Comparison with the state-of-the-art methods

In this section, we compare our proposed networks with 23 state-of-the-art methods, including PCF^5^, MMCI^32^, CPFP^33^, DRMA^8^, D3Net^9^, UCNet^4^, SSF^34^, S2MA^24^, CoNet^35^, cmMS^36^, DANet^23^, A2dele^37^, PAGR^20^, DFM^38^, DSA2f^39^, HAINet^40^, SSL^41^, DisenFuse^42^, ICNet^43^, CMWNet^44^, BBSNet^1^, CDNet^45^ and DCF2^46^. Quantitative and visual comparisons are taken into accounts for fair comparisons.

### Quantitative comparisons

Table 1 shows quantitative comparisons with 23 salient detectors from three perspectives. First and foremost, evaluation scores of all methods on four benchmark datasets present as columns. It is obviously that our models achieve the top-3 performance on NLPR, STERE and SIP for four evaluation metrics. More importantly, our proposed method possesses the least MAE on NLPR and STERE, with approximately 8.7% and 2.7% promotion, respectively. Secondly, we count on the top-3 number of every method. The statistical result is demonstrated in the column named Top 3. It is remarkable that our proposed method occupy the largest number, with 11/16. Finally, we calculate the average value of the evaluated metric on four datasets, which is listed in the row named “Average-Metric”. Our model reach the top-3 performance on all datasets And rank 1st in the average MAE.

**Table 1:** Quantitative comparisons.

	NJUD datasets				NLPR datasets				STERE datasets				SIP datasets			
	MAE	FM	SM	EM	MAE	FM	SM	EM	MAE	FM	SM	EM	MAE	FM	SM	EM
PCF	0.059	0.872	0.877	0.924	0.044	0.841	0.874	0.925	0.064	0.86	0.875	0.925	0.071	0.838	0.842	0.901
MMCI	0.079	0.852	0.858	0.915	0.059	0.815	0.856	0.913	0.068	0.863	0.873	0.927	0.086	0.818	0.833	0.897
CPFP	0.053	0.877	0.879	0.926	0.036	0.867	0.888	0.932	0.051	0.874	0.879	0.925	0.064	0.851	0.85	0.903
DMRA	0.051	0.886	0.886	0.927	0.031	0.879	0.899	0.947	0.066	0.847	0.835	0.911	0.085	0.821	0.806	0.875
D3Net	0.041	0.9	0.9	0.95	0.025	0.897	0.912	0.953	0.046	0.891	0.899	0.938	0.063	0.861	0.86	0.909
UCNet	0.043	0.895	0.897	0.936	0.025	0.903	0.92	0.956	0.039	0.899	0.903	0.944	0.051	0.879	0.875	0.919
SSF	0.043	0.896	0.899	0.935	0.026	0.896	0.914	0.953	0.044	0.89	0.893	0.936	0.053	0.88	0.874	0.921
S2MA	0.053	0.889	0.894	0.93	0.03	0.902	0.915	0.95	0.051	0.882	0.89	0.932	0.054	0.884	0.878	0.92
CoNET	0.047	0.892	0.895	0.937	0.031	0.887	0.908	0.945	0.04	0.904	0.908	0.948	0.063	0.867	0.858	0.913
cmMS	0.044	0.897	0.9	0.936	0.027	0.896	0.915	0.949	0.042	0.891	0.895	0.937	0.061	0.871	0.867	0.091
DisenFuse	0.052	0.897	0.889	0.914	0.035	0.895	0.9	0.933	0.054	0.887	0.883	0.915	0.068	0.866	0.859	0.899
ICNet	0.051	0.903	0.895	0.901	0.028	0.919	0.922	0.945	0.054	0.897	0.891	0.911	0.063	0.882	0.864	0.903
CMWNet	0.046	0.913	0.903	0.923	0.029	0.913	0.917	0.941	0.043	0.911	0.905	0.93	0.062	0.89	0.867	0.909
BBSNet	0.039	0.926	0.916	0.937	0.026	0.921	0.923	0.948	0.046	0.901	0.896	0.928	0.056	0.892	0.874	0.912
CDNet	0.038	0.919	0.913	0.94	0.024	0.925	0.93	0.954	0.041	0.909	0.903	0.938	0.06	0.888	0.862	0.905
DCF2	0.038	0.917	0.903	0.941	0.023	0.917	0.921	0.956	0.037	0.915	0.905	0.943	0.052	0.9	0.873	0.921
DANet	0.048	0.88	0.891	0.932	0.029	0.903	0.915	0.953	0.048	0.881	0.892	0.93	0.054	0.884	0.878	0.92
A2dele	0.052	0.872	0.868	0.914	0.031	0.875	0.89	0.937	0.043	0.885	0.885	0.935	0.07	0.834	0.829	0.889
PGAR	0.045	0.905	0.906	0.94	0.028	0.898	0.918	0.948	0.044	0.893	0.903	0.936	0.059	0.877	0.875	0.914
DFM-Net	0.042	0.91	0.906	0.947	0.026	0.908	0.923	0.957	0.045	0.893	0.898	0.941	0.051	0.887	0.883	0.926
DSA2F	0.039	0.917	0.904	0.937	0.024	0.916	0.918	0.952	0.039	0.91	0.897	0.942	0.057	0.891	0.862	0.911
HAINet	0.038	0.92	0.909	0.931	0.025	0.917	0.921	0.952	0.038	0.919	0.909	0.938	0.048	0.916	0.886	0.925
SSL	0.038	0.923	0.909	0.939	0.025	0.923	0.922	0.96	0.039	0.914	0.904	0.939	0.046	0.909	0.888	0.927
Ours	0.039	0.914	0.902	0.936	0.022	0.922	0.924	0.958	0.036	0.914	0.905	0.941	0.046	0.904	0.886	0.926
Rank	5	7	10	10	1	3	2	2	1	3	3	5	1	3	2	2

	TOP3	Average metrics														
		MAE	FM	SM	EM											
PCF	0/16	0.060	0.853	0.867	0.919											
MMCI	0/16	0.073	0.837	0.855	0.913											
CPFP	0/16	0.051	0.867	0.874	0.922											
DMRA	0/16	0.058	0.858	0.857	0.915											
D3Net	1/16	0.044	0.887	0.893	0.938											
UCNet	1/16	0.040	0.894	0.899	0.939											
SSF	0/16	0.042	0.891	0.895	0.936											
S2MA	0/16	0.047	0.889	0.894	0.933											
CoNET	2/16	0.045	0.888	0.892	0.936											
cmMS	0/16	0.044	0.889	0.894	0.728											
DisenFuse	0/16	0.052	0.886	0.883	0.915											
ICNet	0/16	0.049	0.900	0.893	0.915											
CMWNet	1/16	0.045	0.907	0.898	0.926											
BBSNet	3/16	0.042	0.910	0.902	0.931											
CDNet	5/16	0.041	0.910	0.902	0.934											
DCF2	7/16	0.038	0.912	0.901	0.940											
DANet	0/16	0.045	0.887	0.894	0.934											
A2dele	0/16	0.049	0.867	0.868	0.919											
PGAR	0/16	0.044	0.893	0.901	0.935											
DFM-Net	4/16	0.041	0.900	0.903	0.943											
DSA2F	1/16	0.040	0.909	0.895	0.936											
HAINet	9/16	0.037	0.918	0.906	0.937											
SSL	10/16	0.037	0.917	0.906	0.941											
Ours	11/16	0.036	0.914	0.904	0.940											
Rank	1	1	3	3	3											

For MAE, the lower, the better. On the contrary, for FM, SM and EM, the higher, the better. The second row from the bottom refers to the evaluated scores of our proposed method and the last row refers to the rank of our proposed method.

### Visual comparisons

**Figure 5:**
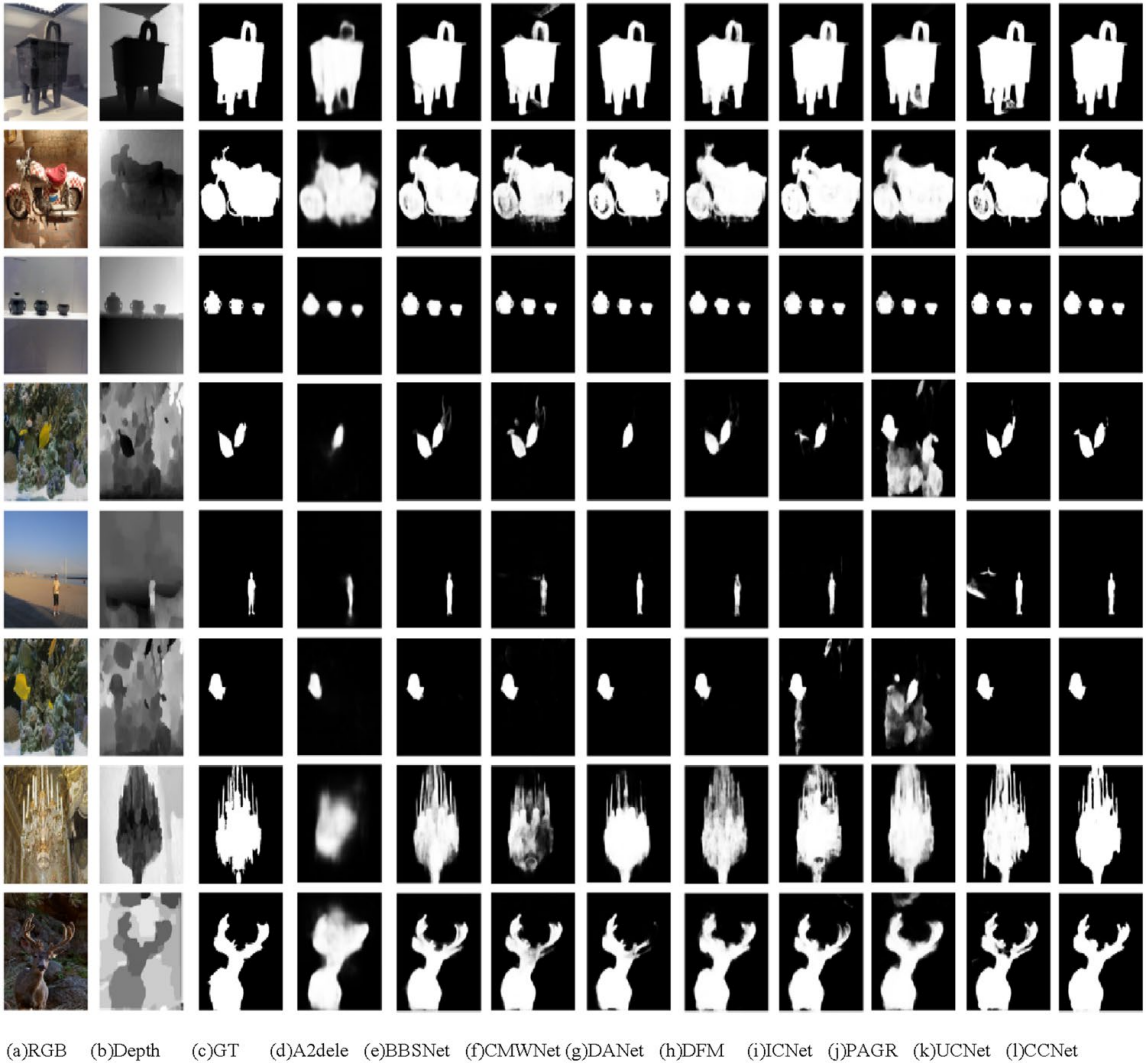
Visual comparisons of different methods. The 1st and 2nd row indicate the complex scenes. The multi-objective object is included in the 3rd and 4th rows. The 5th and 6th mean the scenes of small targets. The low contrast between the background and the object is displayed in 7th and 8th rows. *Note*: Reproduced with permission of references 25, Copyright of ©2017 IEEE, references 26, Copyright of ©2016 IEEE, references 27, Copyright of ©2018 IEEE, references 28, Copyright of ©2015 IEEE.

Figure 5 shows visual comparisons. These examples reflect various scenarios, including complex scenes (1st and 2nd rows), multi-objective salient object (3rd and 4th rows), small objects (5th and 6th rows) and low contrast between salient object and background (7th and 8th rows). All images come from downloading the experimental result from Github directly or training the source codes from the Github and predicting salient object. For complex scenes, the compared approaches mostly predict a blurry salient object and recognize some non-salient part around the salient object as salient part. For the multi-objective detection, several methods miss some salient objects or predict the salient object with noises. When it comes to the small object, the compared methods cannot predict a clear and complete salient object whose size is very small in the image. Lastly, for the scenario of low contrast, the existing salient detectors mostly get poor object smoothness and poor details of the salient object. Besides, some compared methods miss important parts of salient object. To sum up, our proposed method can consistently produce accurate and complete salient maps with sharp edges in various cases.

## Ablation study

In this section, we validate the effectiveness of proposed structures. First and foremost, we evaluate the performance of our proposed FiCaps by comparing it with the U-Net^47^. Furthermore, we testify the strategy of integrating external features with internal capsules in FiCaps. Next, our proposed FDM is evaluated. Finally, the performance of GCM is verified by replacing it with the traditional convolutions. All experimenal results are demonstrated in Table 2.

**Table 2:** Ablation study.

Datasets	NJUD				NLPR				STERE				SIP			
Metrics	MAE	FM	SM	EM	MAE	FM	SM	EM	MAE	FM	SM	EM	MAE	FM	SM	EM
①	0.041	0.910	0.899	0.906	0.023	0.920	0.921	0.957	0.035	0.911	0.905	0.925	0.049	0.900	0.879	0.918
②	0.041	0.909	0.901	0.906	0.026	0.91	0.916	0.948	0.039	0.903	0.9	0.915	0.051	0.894	0.878	0.916
③	0.046	0.907	0.895	0.916	0.031	0.903	0.906	0.941	0.073	0.86	0.82	0.869	0.056	0.897	0.869	0.907
④	0.040	0.911	0.901	0.936	0.022	0.922	0.923	0.957	0.035	0.912	0.903	0.937	0.047	0.902	0.883	0.925
Ours	0.039	0.914	0.902	0.936	0.022	0.922	0.924	0.958	0.036	0.914	0.905	0.941	0.046	0.904	0.886	0.926

The ‘ours’ in Table 2 means our proposed method. The ① refers to the experimental results by replacing the structure of FiCaps with U-Nets. The ② means the experimental results, which FiCaps does not integrate with external features. The ③ indicates the experimental results, extracting and integrating the features of depth image by using the VGG backbone. The ④ refers to the experimental results by replacing the GCM with the traditional convolutions.

### Effectiveness of FiCaps

We evaluate the performance of FiCaps in two aspects. On the one hand, we use the U-Net as the compared structure to evaluate the performance of FiCaps. The FiCaps is replaced with the U-Net and other modules and the parameters remain unchanged. The experimental results in Table 2, the row ① and the row ‘our’, show that our FiCaps outperforms the U-Net. In addition, we evaluate the effectiveness of integrating internal capsules with external features in FiCaps. To verify it, we train our method with and without integrating external features. It is obviously that, from row ② and row ‘our’ in Table 2, the integration of external features is an effective way to improve the performance, with approximately 0.1–9.8% promotions.

### Effectiveness of integration way of depth images

To evaluate the performance of integrating depth images directly, in this section, we try to integrate depth features which are extracted from depth images by VGG, MobileNet^48^ or RESNET18^49^, instead of integrating the depth images directly. In this section, another independent VGG backbone is used to extract depth features from depth images and predict the salient map based on depth images. The extracted depth features are integrated with features from RGB images. The experimental result ③ in Table 2 demonstrates that our proposed method is a more effective way, with about 15.2–50% promotion in MAE and approximately 0.8–2.2% improvement in other evaluation metrics.

### Effectiveness of GCM

To evaluate the contribution of GCM, we replace the GCM with traditional convolutions with batch normalization and Relu operation. The experimental result ④ in Table 2 demonstrates that GCM is an more effective way to integrate features.

## Conclusion

In this paper, we pay much attention to solving the object-part relationship dilemma in the SOD. Therefore, we propose a novel CCNet based on CapsNet with less computation demand, which makes explore the object-part relationship available and applicable. Our proposed method includes two main steps. In the first step, the RGB-D features are extracted and integrated. In the second step, the object-part relationship can be explored fully by using FiCaps. Subsequently, the final salient map is predicted by FiCaps. Extensive experiments on four datasets demonstrate our proposed method outperforms 23 SOTA methods.

More importantly, the FiCaps is transferable for any RGB-D SOD. The FiCaps can be used as a complementary branch for any architecture in the area of SOD to explore the object-part relationship. A feature map is input into the FiCaps and a attention map considering the object-part relationship is predicted. The attention map can be integrated with other features to predict the final map.

In the future, we may focus on two aspects to improve the performance of CCNet. On the one hand, the FiCaps is a convolutional capsule network, to some extent, it is not a pure capsule network. Therefore, as discussed in the related work, the vector CapsNet or the matrix CapsNet may be introduced to explore the object-part relationship in true sense. On the other hand, for reducing the computational demand of CapsNet, mutual learning such as knowledge distillation^50, 51^ may be introduced.

## Data Availability

The data that support the findings of this study are openly available in RGB-D benchmark datasets, including NJU2K^30^, NLPR^31^, STERE^32^ and SIP^33^. They are available on the website https://mmcheng.net/socbenchmark/ or https://www.githubs.cn/projects/272383101-rgbd-sodsurvey.

## References

[1] Fan, D. P., Zhai Y, Ali, B. *et al*. *BBS-Net: RGB-D Salient Object Detection with a Bifurcated Backbone Strategy Network*. https://arxiv.org/abs/02713 (2007).

[2] Fu K, Fan DP, Ji GP, Zhao QJ, Shen JB, Zhu C (2021). Siamese network for RGB-D salient object detection and beyond. IEEE Trans. Pattern Anal. Mach. Intell..

[3] Pang, Y. W., Zhang, L. H., Zhao, X. Q. *et al*. Hierarchical dynamic filtering network for RGB-D salient object detection. In *European Conference on Computer Vision* 235–252 (2020).

[4] Zhang, J., Fan, D. P., Dai Y. C. *et al*. UC-Net: Uncertainty inspired RGB-D saliency detection via conditional variational autoencoders. In *IEEE Conference on Computer Vision and Pattern Recognition* (2020).

[5] Chen, H. & Li, Y. Progressively complementarity-aware fusion network for RGB-D salient object detection. In *IEEE Conf. Comput. Vis. Pattern Recog.* 3051–3060 (2018).

[6] Chen H, Li YF, Su D (2019). Multi-modal fusion network with multiscale multi-path and cross-modal interactions for RGB-D salient object detection. Pattern Recognit..

[7] Zhao, H. S., Shi, J., Qi, X. J., Wang, X. G. & Jia, J. Pyramid scene parsing network. In *CVPR* 6230–6239 (2019).

[8] Piao, Y., Ji, W., Li, J. *et al*. Depth-induced multi-scale recurrent attention network for saliency detection. In *IEEE international conference on computer vision* 7254–7263 (2019).

[9] Fan DP, Lin Z, Zhang Z (2020). Rethinking RGB-D salient object detection: Models, datasets, and large-scale benchmarks. IEEE Trans. Neural Networks Learn. Syst..

[10] Hinton, G. E., Krizhevsky, A. & Wang, S. D. Transforming autoencoders. In *International Conference on Artificial Neural Networks* 44–51 (2011).

[11] Sabour S, Frosst N, Hinton GE (2017). Dynamic routing between capsules. Neural Inf. Process. Syst..

[12] Hinton, G. E., Sabour, S. & Frosst N. Matrix capsules with EM routing. In *International conference on learning representations (ICLR)* 3856–3866 (2018).

[13] Chen, Z., Xu, Q. & Cong, R. Global context-aware progressive aggregation network for salient object detection. In *AAAI Conference on Artificial Intelligence*, Vol. 34, No. 7, 10599–10606 (2020).

[14] LaLonde, R. & Bagci, U. Capsules for object segmentation. *Computer Vision and Pattern Recognition. Machine Learning*. 10.48550/arXiv.1804.04241 (2017).

[15] Woo, S., Park, J. & Lee, J. Y. In So Kweon. Cbam: Convolutional block attention module. In *Proceedings of the European Conference on Computer Vision (ECCV)* 3–19 (2018).

[16] Fu, J., Liu, J., Tian, H. J. *et al*. Dual attention network for scene segmentation. In *IEEE/CVF Conference on Computer Vision and Pattern Recognition* (2019).

[17] Ali B, Cheng MM, Hou QB (2019). Salient object detection: A survey. J. Comput. Vis. Media.

[18] Cong R, Lei J, Fu HJ, Hou JH, Huang QM, Kwong S (2019). Going from RGB to RGBD saliency: A depth-guided transformation model. IEEE Trans. Cybern..

[19] Liang FF, Duan LJ, Ma W, Qiao YH, Cai Z, Qing LY (2018). Stereoscopic saliency model using contrast and depth-guided-background prior. Neurocomputing.

[20] Xu T, Zhao W, Cai L (2023). Lightweight saliency detection method for real-time localization of livestock meat bones. Sci. Rep..

[21] Zhang, X. N., Wang, T. T., Qi, J. J. *et al*. Progressive attention guided recurrent network for salient object detection. In *IEEE Conference on Computer Vision and Pattern Recognition (CVPR)* 714–722 (2018).

[22] Lei X, Cai X, Lu L (2023). SU2GE-Net: A saliency-based approach for non-specific class foreground segmentation. Sci. Rep..

[23] Zhao, X., Zhang, L., Pang, Y. *et al*. A single stream network for robust and real-time RGB-D salient object detection. In *Computer Vision–ECCV 2020: 16th European Conference, Glasgow, UK*, Vol. 22, No. 16, 646–662 (2020).

[24] Liu Y, Zhang DW, Zhang Q (2022). Part-object relational visual saliency. IEEE Trans. Pattern Anal. Mach. Intell..

[25] Chen, X., Zheng, A., Li, J. *et al*. Look, perceive and segment. Finding the salient objects in images via two-stream fixation-semantic cnns. In *IEEE International Conference on Computer Vision* (2017).

[26] Qu LQ, He SF, Zhang JW (2016). RGBD salient object detection via deep fusion. IEEE Trans. Image Process..

[27] Han JW, Chen H, Liu N (2018). CNNs-based RGB-D saliency detection via cross-view transfer and multiview fusion. IEEE Trans. Cybern..

[28] Tao D, Cheng J, Song M, Lin X (2015). Manifold ranking-based matrix factorization for saliency detection. IEEE Trans. Neural Netw. Learn. Syst..

[29] Achanta, R., Hemami, S., Estrada, F. *et al*. Frequency-tuned salient region detection. In *IEEE International Conference on Computer Vision and Pattern Recognition* 1597–1604 (2009).

[30] Fan, D. P., Cheng, M. M., Liu, Y. *et al*. Structure measure: A new way to evaluate foreground maps. In *IEEE International Conference on Computer Vision* 4548–4557 (2017).

[31] Fan, D. P., Gong, C., Cao, Y. *et al*. Enhanced-alignment measure for binary foreground map evaluation. In *International Joint Conference on Artificial Intelligence* 698–704 (2018).

[32] Chen H, Li YF, Su D (2019). Multi-modal fusion network with multi-scale multi-path and cross-modal interactions for RGB-D salient object detection. Pattern Recognit..

[33] Zhao, J. X., Cao, Y, Fan, D. P. *et al*. Contrast prior and fluid pyramid integration for RGBD salient object detection. In *IEEE Conference on Computer Vision and Pattern Recognition* (2019).

[34] Zhang, M., Ren, W., Piao, Y. *et al*. Select, supplement and focus for RGB-D saliency detection. In *IEEE/CVF conference on computer vision and pattern recognition* 3472–3481 (2018).

[35] Ji, W., Li, J., Zhang, M. *et al*. Accurate RGB-D salient object detection via collaborative learning. In *Computer Vision–ECCV 2020: 16th European Conference, Glasgow, UK*, Vol. 18, No. 16, 52–69 (2020).

[36] Li, C., Cong, R., Piao, Y. *et al*. RGB-D salient object detection with cross-modality modulation and selection. In *Computer Vision–ECCV 2020: 16th European Conference, Glasgow, UK*, Vol. 8, No. 16, 225–241 (2020).

[37] Piao, Y., Rong, Z., Zhang, M. *et al*. A2dele: Adaptive and attentive depth distiller for efficient RGB-D salient object detection. In *IEEE/CVF Conference on Computer Vision and Pattern Recognition* 9060–9069 (2020).

[38] Zhang, W., Ji, G. P., Wang, Z. *et al*. Depth quality-inspired feature manipulation for efficient RGB-D salient object detection. In *29th ACM International Conference on Multimedia* 731–740 (2021).

[39] Sun, P., Zhang, W. H., Wang, H. Y. *et al*. Deep RGB-D saliency detection with depth-sensitive attention and automatic multi-modal fusion. In *CVPR* 1407–1417 (2021).

[40] Li GY, Liu Z, Chen MY (2021). Hierarchical alternate interaction network for RGB-D salient object detection. IEEE Trans. Image Process..

[41] Zhao, X. Q., Pang, Y. W., Zhang, L. H. *et al*. *Self-Supervised Representation Learning for RGB-D Salient Object Detection*. 10.48550/arXiv.2101.12482 (2021).

[42] Chen H, Deng YJ, Li YF (2020). RGBD salient object detection via disentangled cross-modal fusion. IEEE Trans. Image Process..

[43] Li GY, Liu Z, Ling HB (2020). ICNet: Information conversion network for RGB-D based salient object detection. IEEE Trans. Image Process..

[44] Li, G., Liu, Z., Ye, L. *et al*. Cross modal weighting network for RGB-D salient object detection. In *ECCV* 665–681 (2020).

[45] Jin WD, Xu J, Han Q (2021). CDNet: Complementary depth network for RGB-D salient object detection. IEEE Trans. Image Process..

[46] Ji, W., Li, J. J., Yu, S. *et al*. Calibrated RGB-D salient object detection. In *CVPR* 9471–9481 (2021).

[47] Man N, Guo S, Yiu KFC (2023). Multi-layer segmentation of retina OCT images via advanced U-net architecture. J. Neurocomput..

[48] Howard, A. G., Zhu, M. & Chen, B. MobileNets: Efficient convolutional neural networks for mobile vision applications (2017).

[49] He, K., Zhang, X. & Ren, S. Deep residual learning for image recognition. In *2016 IEEE Conference on Computer Vision and Pattern Recognition* (2016).

[50] Chen, P. G., Liu, S., Zhao, H. S. & Jia, J. Y. Distilling knowledge via knowledge review. In *CVPR* (2021).

[51] Li, Z., Ye, J., Huang, Y. & Pan, Z. Online knowledge distillation for efficient pose estimation. In *ICCV* (2021).

